# In the Wild HRI Scenario: Influence of Regulatory Focus Theory

**DOI:** 10.3389/frobt.2020.00058

**Published:** 2020-04-30

**Authors:** Roxana Agrigoroaie, Stefan-Dan Ciocirlan, Adriana Tapus

**Affiliations:** Autonomous Systems and Robotics Laboratory, U2IS, ENSTA-Paris, Institut Polytechnique de Paris, Palaiseau, France

**Keywords:** HRI, regulatory focus, in the wild, acceptance, social robotics

## Abstract

Research related to regulatory focus theory has shown that the way in which a message is conveyed can increase the effectiveness of the message. While different research fields have used this theory, in human-robot interaction (HRI), no real attention has been given to this theory. In this paper, we investigate it in an in the wild scenario. More specifically, we are interested in how individuals react when a robot suddenly appears at their office doors. Will they interact with it or will they ignore it? We report the results from our experimental study in which the robot approaches 42 individuals. Twenty-nine of them interacted with the robot, while the others either ignored it or avoided any interaction with it. The robot displayed two types of behavior (i.e., promotion or prevention). Our results show that individuals that interacted with a robot that matched their regulatory focus type interacted with it significantly longer than individuals that did not experience regulatory fit. Other qualitative results are also reported, together with some reactions from the participants.

## 1. Introduction

It is a well-known fact since ancient times that people approach pleasure and avoid pain. Looking at this from a different perspective, we can imagine that people approach or engage in tasks which they find enjoyable, and avoid tasks or situations which brings them pain, or that they do not find enjoyable. While not all tasks that individuals have to perform in their every-day working life can be viewed as only enjoyable or not enjoyable, the question arises at to which strategies will they apply in order to achieve their goals? In the psychology literature, Higgins (1997) introduces a theory stating that individuals adopt one of two possible approaches in achieving a goal.

The theory is the Regulatory Focus Theory (RFT) (Higgins, 1997) and the two approaches are: promotion and prevention. In Crowe and Higgins (1997), the authors characterize promotion type individuals as individuals that guide their actions toward achieving their goals. Whereas, prevention type individuals guide their actions in order to avoid failure.

According to Higgins (2000), regulatory fit is defined as an increased motivational intensity that is experienced when the manner in which an individual engages in an activity sustains his/her current interests. As an example, in order to successfully pass a course, a promotion type individual will be more inclined to read supplementary material in order to maximize their results, while a prevention type individual will be careful to fulfill the minimum course requirements in order to pass.

Furthermore, it was also shown in Higgins (2000, 2005) that people that experience regulatory fit, engage more strongly in their current activity. Therefore, it is expected that individuals who experience regulatory fit will engage for longer in a given task. Moreover, regulatory fit can be used to effectively change attitudes and behaviors, and to improve the quality of life in interpersonal conflicts. For instance, regulatory fit and non-verbal cues can be used to increase persuasiveness (Cesario and Higgins, 2008) (e.g., body gestures, movement speed, or speech rate).

Robots are more and more present in our every-day lives. As a result, more research is being carried in which robots play a social role in human-centric environments. Their roles can be diverse, ranging from a teacher for children (Tazhigaliyeva et al., 2016), to personal companion (Breazeal, 2017). By taking into consideration the regulatory focus theory and the more and more social role of robots, we can imagine that robots have the potential of helping individuals to achieve their goals, to increase their motivation (Nakagawa et al., 2011; Andrist et al., 2015).

The authors of Faur et al. (2015) have designed artificial agents based on RFT. They designed a game scenario and have found that regulatory fit had an effect on the prevention type end-users (i.e., likability of the game). The RFT was also successfully used in designing persuasive technologies (Rezai et al., 2017). On the other hand, a review of the HRI literature has shown that RFT has not received much attention. The first study using RFT in an HRI scenario is presented in Cruz-Maya et al. (2017). The authors have improved the performance of the participants in a Stroop task, by matching the behavior of the robot with the regulatory focus type of the participants. The same authors, have continued to use of RFT in a negotiation type scenario with a humanoid robot (Cruz-Maya and Tapus, 2018). Their results show that RFT and regulatory fit can be successfully used in HRI scenarios.

The study presented in this paper is based on the works of Cruz-Maya et al. (2017); Cruz-Maya and Tapus (2018). Thus, the purpose of this study is to investigate if RFT can be applied in an in the wild HRI scenario. Our main research question is **RQ: “How do individuals (based on their RFT type) react when a robot appears at their doorway to ask them to perform a short questionnaire?”**. By applying different strategies (either promotion or prevention) we wanted to investigate if individuals will be more inclined to perform the task. Of interest for this study is not the answers given to the questionnaire, but if the participants approached the robot to interact with it or they just ignored it based on the robot's behavior and user's regulatory focus type (promotion or prevention type).

The paper is structured as follows. Section 2 is dedicated to the presentation of the interaction scenario, robot navigation, and robot behavior. The results are presented in section 3, while section 4 shows a discussion of these results. Lastly, the paper is concluded in section 5.

## 2. Methodology

This study is designed as a 2 (behavior of robot, i.e., promotion or prevention) × 2 (regulatory focus type of the participants) between participants experimental study. In Table 1 is presented the distribution of the participants into the four conditions.

**Table 1:** Distribution of participants based on different factors.

**Knowledge about robotics**				
1 (“Not at all”)	2	3	4	5 (“Very much”)
4	4	9	6	6
**Regulatory focus results**				
Promotion			Prevention	
19			10	
**Conditions**					
Robot	Participant			
	Promotion	Prevention		
Promotion	11	6		
Prevention	8	4		

According to Higgins (2000, 2005), individuals who experience regulatory fit engage more strongly in the activity they are performing. Therefore, we hypothesize that the participants that experience regulatory fit will engage for longer with the robot than the participants that do not experience regulatory fit.

**H: Participants that have a matching regulatory focus type with the behavior of the robot (i.e., regulatory fit) will interact with the robot for longer than the participants that do not have a matching regulatory focus type with the behavior of the robot**.

Taking into account the different office layouts and the different times needed by the participants to reach the robot, we consider the measure **time_interaction** as the time needed by the participants to fill in the questionnaire (i.e., between pressing the **START** and **QUIT** buttons) and it represents the interaction time between the participants and the robot.

To test our research hypothesis, we consider the measure time_interaction as the *dependent variable*, and the regulatory fit/no fit as the *independent measure*.

### 2.1. Scenario

**Figure 1:**
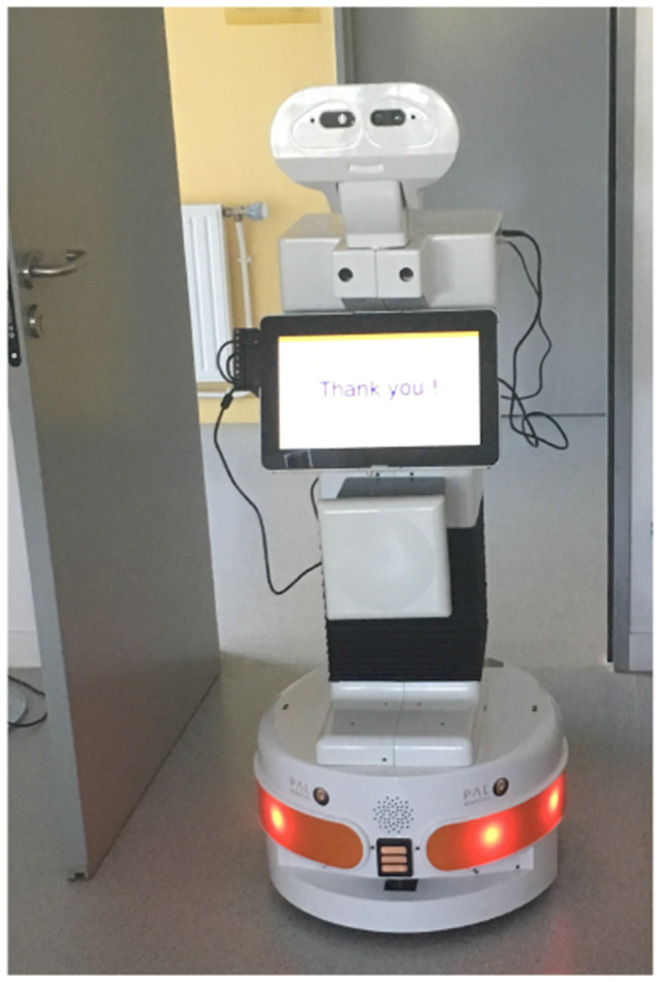
Tiago robot.

For this study, Tiago, a robot developed by PAL Robotics^1^, was used. The robot features a mobile base, a lifting torso, a touch screen mounted on its torso (as shown in Figure 1), and a head. The eyes of the robot are equipped with an RGB-D camera and the speakers are located between the head of the robot and the touch-screen.

**Figure 2:**
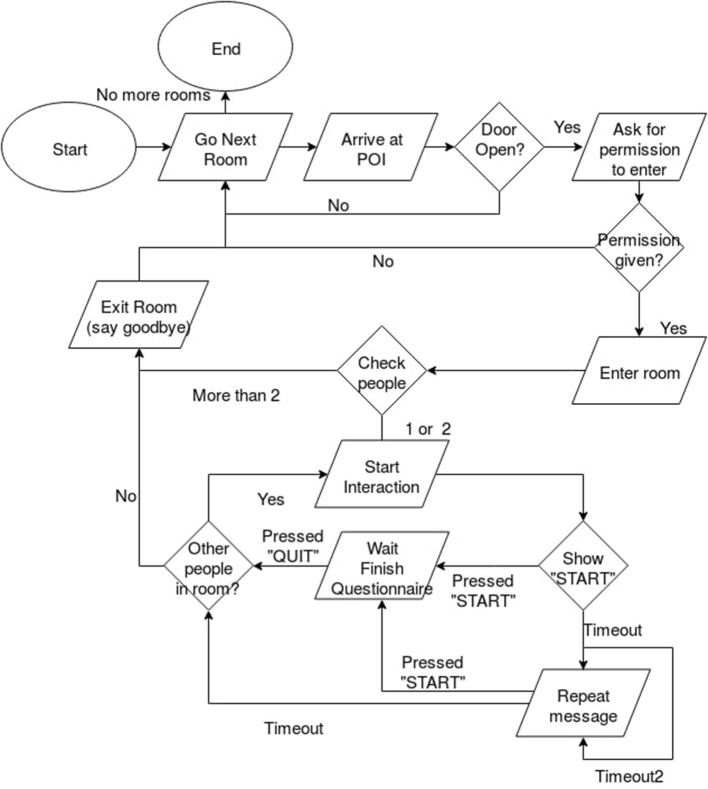
Experimental scenario.

The study presented in this paper was carried out at the university where the authors are located. The participants are some of the employees from the various departments of the university and they were not informed in any way about this study. The diagram of the experimental scenario is shown in Figure 2.

The experiment starts with the robot loading the map (see section 2.2) and the points of interest (POI) corresponding to the doorways of the offices in the university. Each POI had a corresponding flag that indicated if the office was visited before or not. The robot started with the first POI and then continued to visit each office, until all offices were visited.

Once arrived at a POI, by using its laser, the robot checked if the current door was open. However, due to frequent laser malfunctions, this information was given to the robot by the investigators. Even if the robot was navigating autonomously, the investigators were always in the close proximity of the robot, just to make sure that there were no problems during the interaction. Moreover, the investigators made sure that the participants did not see them.

Next, the robot started checking how many people were in the office. For small interaction distances (i.e., <1.5 m), the robot is able to accurately detect how many people are in an office by using the face detector provided by the Dlib toolkit (King, 2009). However, since there were no two offices with the same layout, and the lightning conditions were very different from one office to another, using an automatic face detector proved not to be very reliable for this scenario. Therefore, as a fail-safe method, we decided to manually determine how many people were in an office, by checking the video-feed provided by the RGB-D camera located in the eyes of the robot.

The interaction was designed for at most two people in an office. If more than two people were detected, the robot would turn around and leave the office. Otherwise, it randomly chose its behavior (i.e., either promotion or prevention) and it would say the message presented in section 2.3. It had a waiting time of 30 s (Timeout, in Figure 2). This moment is considered as the first ping (i.e., the first time that the person hears the message from the robot). If during the first waiting time there was no reaction from the person, the robot approached the desk and it would repeat the same message after saying “*Excuse me, can you please listen to me?”*. This moment is considered as the second ping (i.e., the second time that the person hears the message). When the robot approached the desk it would use a moving speed appropriate to the behavior that is currently displaying (see section 2.3). Then, it would wait for 15 s (Timeout2), and if there was no reaction from the person, it would say again the same message (i.e., third ping) and waited for another 30 s (Timeout) for a reply. If the participant still did not want to interact with the robot, it thanked the person and approached the second person in the office (if there was one) or it left the office. When leaving the office the robot set the flag for the office as visited and it approached the next office.

**Figure 3:**
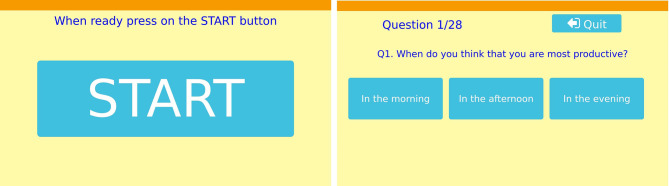
**(Left)** Start button for starting the questionnaire; **(Right)** Example question.

By reaction from the person it is understood that the person would approach the robot and press on the **START** button displayed on the tablet of the robot [see Figure 3 (left)]. The participants could stop at any time by pressing on the **Quit** button located on the upper right corner of the screen [see Figure 3 (right)]. The participant could see at all times the number of the current question and the total number of questions.

The task that the participants had to perform, was a 28 questions questionnaire that was displayed on the tablet of the robot. The questions concerning stress at work were selected from the Copenhagen Psychosocial Questionnaire (COPSOQ) (Kristensen et al., 2005). The dimensions selected from the questionnaire were: Cognitive demands (e.g., “Does your work require you to make difficult decisions?”), Work engagement (e.g., “I am enthusiastic about my job”), Stress (e.g., “How often have you had problems relaxing?”), Cognitive stress (e.g., “How often have you had problems concentrating?”), and Self-efficacy (e.g., “I feel confident that I can handle unexpected events”). Some other questions were added that were not part of the questionnaire (e.g., “Do you have enough time in a day to complete your work?”).

### 2.2. Robot Navigation

A total of four maps were created of the entire environment. Each floor of the school contains offices as well as laboratories and small classrooms. We created maps only for the office regions on each floor. For this purpose, the advanced navigation system designed by Pal Robotics was used. The navigation module is based on the ROS (Quigley et al., 2009) 2D navigation stack^2^.

**Figure 4:**
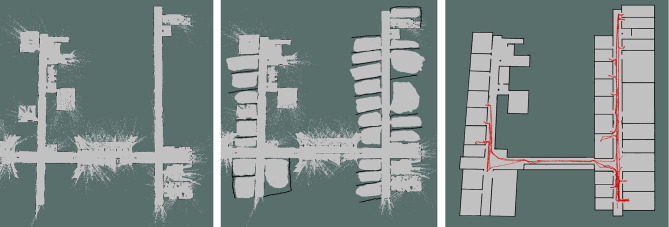
**(Left)** Map created by the robot; **(Middle)** Map used for navigation; **(Right)** Example of one of the paths of the robot overlayed on one of the modified maps.

The navigation software can be used to perform the mapping as well as to enable the robot to autonomously navigate on the selected map. The mapping system of the robot (i.e., GMapping^3^) uses the readings from the 2D laser scanner which is located on the mobile base to create an Occupancy Grid Map (OGM). Usually when there is somebody in an office, the office door is left open. Therefore, as the mapping was done after the usual working hours, most of the offices were already closed (see Figure 4 (left) for the original map). Therefore, the map was modified in GIMP^4^ so as to contain the approximate shape of the offices [see Figure 4 (middle)]. Otherwise, the robot did not know about the existence of the office and the global planner would not allow the robot to enter the office. This modified map was used by the robot to localize itself and to navigate autonomously in the environment. We used the local and the global planners designed by Pal Robotics.

The doorway of each office was designated as a POI. The robot could then easily plan its path from its current location to any of the POIs defined on the map. For each office, we also defined a secondary POI immediately inside the office, in front of the door. This enabled the robot to easily enter each office. At the end of each interaction the robot navigates to the primary POI of the office and then to the POI of the next office door. As the investigators were always in the close proximity of the robot, in case the robot's local planner would not find a possible path and detect that it could not exit the office, they controlled the robot to reach the doorway and then let the robot autonomously move to the next POI. The intervention of the experimenters was required only for a few instances. In the majority of the interactions, the robot successfully found a path for it to exit the office and to reach the next POI.

Next, the behavior of the robot is presented.

### 2.3. Robot Behavior

As shown in section 1, the way in which a message is conveyed can increase the effectiveness of that message. Therefore, of importance is the way in which the message is framed by the robot in order to persuade the participants to stop whatever they were doing and to start an interaction with it.

The robot could display one of two behaviors (i.e., promotion type or prevention type). As shown in Lee and Aaker (2004), a regulatory fit can be created by using an eager framing for promotion type individuals, and by using a vigilant framing for prevention type individuals. The research presented in Cesario and Higgins (2008) describes which non-verbal cues can be used to define a vigilant and an eager type behavior. Thus, an eager type behavior is characterized by: *fast body movement, fast speech rate, and open hand movements*, among others. On the other hand, a vigilant type behavior is characterized by: *slower body movement, a slower speech rate, and by gestures that show precision*, among others. As our robot does not feature any arms, we could only change the body movement and the speech rate between the two behaviors. Moreover, we also changed the speed of the approach accordingly. No indication was found in the literature for the specific values for the speech rate and the approach speed for the robot. They were empirically set by the experimenters, by taking into consideration also some of the hardware limitations of the robot. Therefore, as the maximum speed of the robot is of 1 m/s, we decided to select an approach speed of 0.6 m/s for the promotion type robot, and an approach speed of 0.2 m/s for the prevention type robot. While preparing the study, the robot did not have a French TTS engine, thus we used a different TTS^5^ engine to generate the audio files that contained the speech of the robot. From the online TTS engine, we chose the slow speech rate for the prevention robot (which corresponds to a speech rate of ~150 words/min) and the fast speech rate for the promotion robot (which corresponds to a speech rate of ~198 words/min). For each interaction the robot randomly selected between the two types of behavior (i.e., promotion or prevention).

Another important aspect to be considered is how the message is presented. More specifically, the framing of the message can show the recipient the desirable or the undesirable outcomes from successfully or unsuccessfully pursuing a certain goal (Higgins, 2005). Therefore, for the promotion type behavior, the message had to be framed so as to show what was the desirable outcome for the robot if the individual successfully pursuits the task asked by the robot. However, for the prevention type behavior, the message had to be framed in such a manner as for the individual to understand which was the undesirable outcome if he/she does not successfully pursuit the task asked by the robot. Based on these considerations, we designed the following messages and non-verbal cues:

#### 2.3.1. Promotion Type Robot

The robot had a moving speed of 0.6 m/s and the speech rate of 198 words/min, with a total speech time of 18 s. The message was the following:

Hello. My name is Tiago. I am trying to learn more about stress at the workplace. I have 28 questions for you. **If you answer at least 20 of them, I will be able to learn more about what it is like to be active in the workforce**. You can stop at anytime you want by pressing on the QUIT button. When you are ready you can press on the START button.

#### 2.3.2. Prevention Type Robot

The speed of the robot was set to 0.2 m/s and the speech rate of 150 words/min, with a total speech time of 25 s. The message was the following:

Hello. My name is Tiago. I am trying to learn more about stress at the workplace. I have 28 questions for you. **If you do not** **answer at least 20 of them, I will not** **be able to learn more about what it is like to be active in the workforce**. You can stop at anytime you want by pressing on the QUIT button. When you are ready you can press on the START button.

### 2.4. Participants

For this experiment, a total of 42 participants were approached by the robot. Out of these, 29 (69%) interacted with it, while the others either avoided it completely or simply ignored it. At the end of the experiment, the 29 participants (8 female and 21 male) that interacted with Tiago, signed a consent form that allows us to use their data for research purposes. Moreover, they were also asked to fill in the questionnaires presented at the end of this section, and to answer some demographic questions. The ages of the participants ranged between 23 and 52 years old (*M* = 36.42, *SD* = 9.86). When asked about their background, 14 of them had a computer science background, 7 had a technical background, while for the other participants their backgrounds were diverse, including, linguistics, statistics, human resources, or art history. All the participants were asked to rate their knowledge about robotics, on a scale from 1 (“Not at all”) to 5 (“Very much”). The results are shown in Table 1. Even if the majority of the participants (17 out of 29) had no serious knowledge about robotics, 25 of them interacted with a robot before.

To determine the regulatory focus type of the participants, the Regulatory Focus Questionnaire—proverb form (Faur et al., 2017) was given to each of them upon the completion of the experiment. Therefore, the experimenters did not know before the interaction the regulatory type of the participants. The questionnaire contains 18 proverbs and it was originally developed in French. The proverbs were translated into English for the 6 participants that were not French native. The distribution of the participants is shown in Table 1.

From the BIG5 (Goldberg, 1990) personality questionnaire only the questions related to the Conscientiousness personality trait were selected. As shown in the review paper (Barrick and Mount, 1991), research has shown that an individual with high conscientiousness is dependable, hard-working, persevering. Therefore, we believe that the level of conscientiousness will influence the number of questions answered during the interaction.

The last questionnaire that the participants had to fill was a custom designed post-questionnaire, in which the participants were asked to rate, on a Likert scale from 1 (“Strongly Disagree”) to 5 (“Strongly Agree”) their thoughts about the robot's behavior (e.g., polite, persuasive, motivating, intimidating).

## 3. Results

### 3.1. Hypothesis Results

As previously shown, our research tries to show that participants will interact for longer with a robot that displays a behavior that matches their regulatory focus type than with a robot that displays a behavior that does not match their regulatory focus type.

The two assumptions for the ANOVA analysis are the normal distribution of the data and the variance across groups has to be homogeneous. First, we tested the normal distribution of the data by applying a Shapiro-Wilk normality test. With a *p*-value > 0.05 (*W* = 0.94, *p* = 0.16), we can conclude that our data is normally distributed. Next, we apply Levene's test for homogeneity of variance across groups. Based on our results, [*F*_(1, 27)_ = 1.79, *p* = 0.19] we can assume the homogeneity of variances in the two groups.

**Figure 5:**
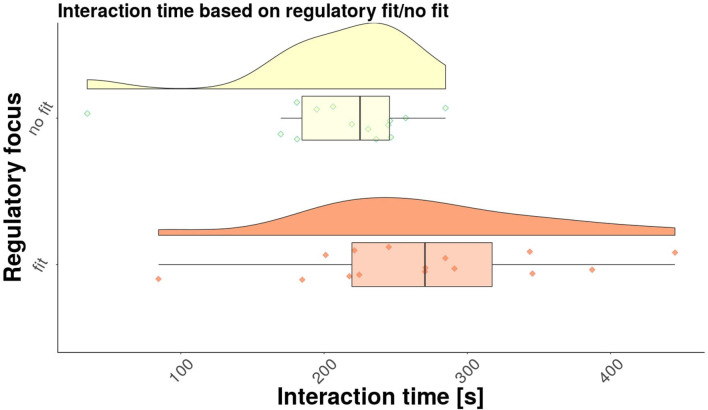
Raincloud plot for interaction time based on regulatory fit/no fit.

Therefore, we can apply one-way ANOVA analysis to test our hypothesis. The results of the test, as well as the summary statistics by groups (i.e., count, mean, standard deviation) are presented in Table 2. Based on these results, we can conclude that our **research hypothesis is validated**. The participants that interacted with a robot that matched their regulatory focus type interacted with it for longer than the participants that interacted with a robot that did not match their regulatory focus type. This result is also represented graphically as a raincloud plot^6^ in Figure 5. To further validate our results we have performed a power analysis for the one-way analysis of variance by applying the specific function from the **pwr** R package that implements the power analysis as outlined by Cohen (2013). With the two groups (fit, no fit), a common sample size in each group of 14 participants and a power of 0.8, and a significance level of 0.05, our results show that the effect size for our one-way ANOVA analysis is equal to 0.55. According to Cohen (2013), this result represents a large effect size.

**Table 2:** Results for research hypothesis.

**Summary statistics by groups**			
**Group**	**Count**	**Mean**	**SD**
Fit	15	268	88.7
No Fit	14	210	60.2
**Anova results**			
	**Df**	**F**	**Pr (>F)**
Fit	1	4.21	0.049^*^
Residuals	27		

* *Represents the standard way of representing a significant result for a p-value less than 0.5*.

*Bold values indicates that the result is significant*.

**Figure 6:**
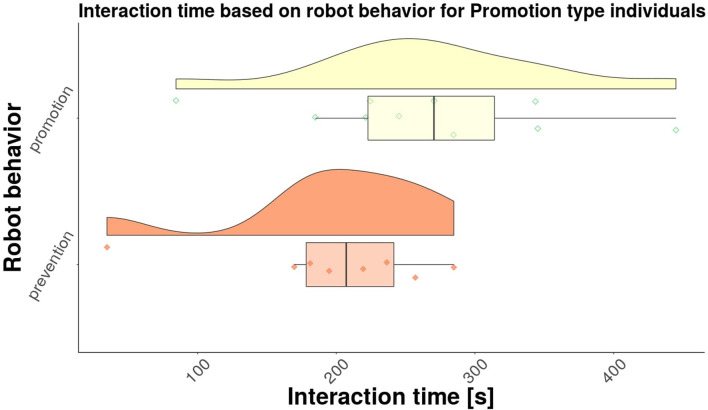
Interaction times for promotion type individuals.

**Figure 7:**
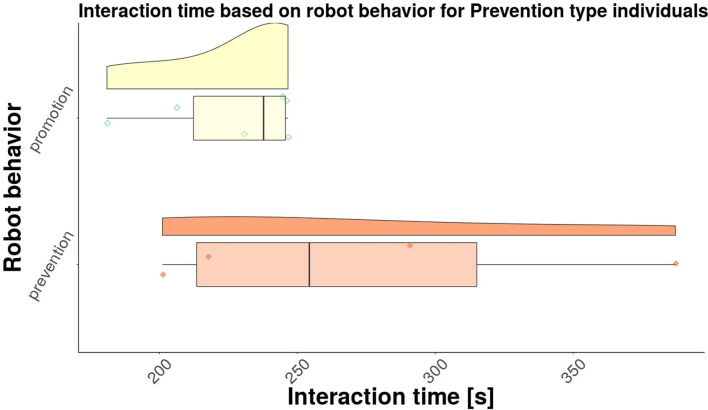
Interaction times for prevention type individuals.

Next, we investigated separately the results for the Promotion type individuals, as well as for the Prevention type individuals. Raincloud plots were created for each group, as shown in Figure 6 for promotion type individuals, and in Figure 7 for the prevention type individuals, respectively. The average interaction times for each group are shown in Table 3. While it is clear from the table that the participants that experienced regulatory fit interacted longer than participants that did not experience regulatory fit, the differences between the two robot behaviors are not significant [*F*(1, 17) = 2.83, *p* = 0.11 for promotion type individuals, and *F*(1, 17) = 1.78, *p* = 0.22 for prevention type individuals, respectively]. Further investigation is needed in order to determine if there are significant differences between the promotion and prevention types individuals.

**Table 3:** Interaction times for each group.

**Interaction times (s)**		
	**Promotion type Mean (SD)**	**Prevention type Mean (SD)**
Promotion behavior	265.42 (94.05)	225.90 (26.89)
Prevention behavior	197.25 (76.27)	274.24 (84.79)

### 3.2. Qualitative Results

We believe it is also noteworthy to present a selection of the qualitative results. They provide valuable insight into how individuals react when they are approached by a robot, without being told beforehand. We consider as qualitative results some of the reactions of the individuals that either interacted, avoided, or ignored the robot. First, we present examples of the reactions of the individuals that did not interact with the robot.

**Figure 8:**
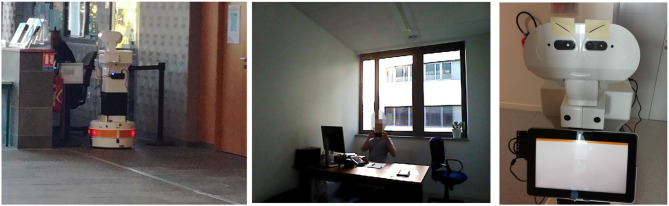
**(Left)** Individual just looking at the robot without interacting; **(Middle)** Participant filming Tiago; **(Right)** One participant gave Tiago some eye-brows.

A total of 13 individuals (5 females and 8 males) refused to interact with Tiago. One individual completely ignored it, by putting back his headphones. Other two individuals just looked at the robot while it talked to them, but did not display any intention of interaction [see Figure 8 (left)].

Two individuals first wanted to interact with the robot, however, they had to leave their offices due to work obligations. One individual came to our office and approached us to ask if it is necessary for her to interact with the robot, as she was very busy. We tried to limit the interaction with her as much as possible by telling her that it is totally optional and that it is her choice if she wants to interact with the robot or not.

In one office, the occupants were very angry when they saw the robot in their doorway. They thought that the robot was very invasive and they demanded for the robot to be removed from their office door. As the investigators were seeing and hearing remotely the reaction of these participants, the robot was remotely controlled to say “*Bye Bye! Thank you for the interaction*” and to leave the office.

Another interesting reaction from the individuals that avoided the robot consisted of closing the office when the robot approached. For example, two individuals (from two different offices) showed real interest when they saw the robot in the hallway. However, when the robot tried to approach their doors, they shut the door clearly showing that they had no interest to interact with it.

From the interactions with the 29 participants there were a couple of unexpected reactions. One participant when seeing the robot, put two post-its on the head of the robot representing the eye-brows [see Figure 8 (right)]. Other participants were very excited and started taking pictures of the robot or even filming it [see Figure 8 (middle)].

There were participants that were very serious while filling the questionnaire, while others smiled and continuously spoke with Tiago. One female participant saw the robot in the hallway and she started talking to it and saying things like, “*Come and follow me Tiago. I want to interact with you*.” She saw that the name Tiago was written on the back of the robot, so she supposed that the robot is called Tiago.

### 3.3. Other Results

First, we looked at the number of questions answered by the participants. From the 29 individuals that started the questionnaire, 26 answered all questions, one participants answered two questions, one participant answered 4 questions and one participant answered 11 questions. Taking into consideration these results, we do not have enough participants that did not complete the entire questionnaire in order to investigate if the conscientiousness level of the participants had any influence. Of the 29 participants, 28 had a conscientiousness score ≥3 (on a scale from 1 to 5).

Of the total participants, 24 approached the robot after the first ping (i.e., the first time that the robot said the message presented in section 2.3). One participant approached the robot after the second ping (promotion type individual interacting with a promotion type robot), and four participants approached the robot only after the third ping. Again, considering the distribution of the participants based on the number of pings, we cannot investigate further these results.

Next, we were interested in finding out what were the impressions of the participants of the robot. By using a post-questionnaire we assessed on a scale from 1 (“Strongly disagree”) to 5 (“Strongly agree”) if the participants thought that the robot was: polite, friendly, intimidating, motivating and persuasive. In Table 4 are shown the results of the post-questionnaire. The majority of the participants, both in the regulatory fit group, as well as in the regulatory no-fit group, agreed or strongly agreed with the statement that the robot was polite, with an average rating of *M* = 4.24 (*SD* = 0.63). A similar result was observed for the statement that the robot is friendly. Eleven participants in the regulatory fit group either agreed or strongly agreed that the robot is friendly, while 10 participants in the regulatory no-fit group either agreed or strongly agreed with the statement. An average rating of *M* = 3.79 (*SD* = 0.77) was obtained. With an average rating of *M* = 2.51 (*SD* = 1.32), the participants in the study did not agree with the statement that the robot is intimidating. Similar average ratings were found in the regulatory fit group (*M* = 2.4) and in the regulatory no-fit group (*M* = 2.64).

**Table 4:** Distribution of participants based on different factors (1 - “Strongly disagree”; 5 - “Strongly agree”).

	**Regulatory fit**					**Regulatory no-fit**				
	**1**	**2**	**3**	**4**	**5**	**1**	**2**	**3**	**4**	**5**
Polite	–	–	2	10	3	–	–	1	6	7
Friendly	–	1	3	10	1	–	1	3	7	3
Intimidating	5	4	2	3	1	3	4	4	1	1
Motivating	–	5	7	3	–	1	2	6	4	1
Persuasive	1	2	3	7	1	1	2	3	7	1

After completing the experiment, the path of the robot was overlayed on the extended map of each floor. An example of a final result of a map is shown in Figure 4 (right). From the figure it can be seen that the robot moved very much in the area around the lower right corner of the map. This is due to the fact that is the location of the laboratory from where the experiment started.

## 4. Discussion

Even if not all the individuals approached by the robot chose to interact with it, the reaction of all 42 individuals is interesting and needs to be considered. It is quite remarkable that some people would choose to intentionally get up from their desks and to close the door of their offices when the robot tried to interact with them. Even more so, as the participation was completely voluntary. The robot could be ignored, or, as the robot also told them, the interaction could have been stopped at any given time, by simply pressing on the Quit button. Therefore, we consider that this is one of the limitations of this study. It is possible that some of the individuals that chose to deliberately ignore the robot were indeed quite busy and that is why they chose to close the door. Maybe at a different time of the day, or even during a different day they would have been more inclined to interact with the robot. Another aspect to consider is that in some offices there were multiple individuals. This could have influenced if the individuals chose to interact with the robot or not and also the length of the interaction.

When the interaction was over and the investigators approached the participants to give them the questionnaires, some participants stated that when they reached question 20, the number that had to be reached so that the robot can learn more about stress at the work place, they thought about pressing on the Quit button. However, they chose to complete the questionnaire, saying that, as long they started the questionnaire, they might as well finish it.

One results that we found shows that when interacting with a promotion type robot, the participants started approaching the robot faster than when interacting with a prevention type robot. RFT states that increased motivation happens when there is a regulatory fit between the regulatory profile of the person and the behavior of the agent it interacts with (Higgins, 2005). However, in Cesario and Higgins (2008) it was shown that the effectiveness of a message can be increased by using faster rates for conveying a message. This also leads to an increase in the competence and credibility of the source of the message. Therefore, we can conclude, that the participants that interacted with the promotion type robot (i.e., 17 out of 29 interactions), were more eager to approach the robot. This result can be of potential interest for designing HRI scenarios.

Even though, at some moments there was still a need for the investigators to intervene in order to make sure that the interaction was as natural as possible, the robot was autonomous most of the time of the interaction. It has to be taken into consideration that the interaction was carried out in the wild and not in a controlled environment (e.g., in a laboratory). Furthermore, the participants were not aware that they will interact with a robot. They were not previously recruited to take part in the experiment. They were performing their every-day tasks at the workplace and suddenly the robot appeared in their doorway. Thus, the reactions of some individuals are totally understandable (e.g., ignoring the robot, going outside their offices and looking in the hallway to see if they can find the operator of the robot), while others can be considered as surprising (e.g., intentionally going and closing the door).

Further research in the wild is needed in order to better understand how individuals of all ages react toward robots. Of course, these results might have been different if the interaction were to take place with students, with the elderly, or with different groups of individuals. Furthermore, results might have been different in other countries.

One limitation of this study is related to the relatively small number of individuals approached by the robot (i.e., 42 individuals). The RFT was mostly studied in the psychology literature. Therefore, the number of participants in these studies is in the hundreds of participants, while for our study we recruited 42 participants. However, if we consider the related studies in the HRI literature, we can find a similar number of participants as in our study [e.g., in Faur et al. (2015) 20 participants were recruited, while in Cruz-Maya and Tapus (2018) a total of 40 participants took part in the study]. Therefore, we consider that before performing a large scale study, it was important to investigate and to try to understand how individuals might react in such a scenario. Further research is currently planned based on the currently obtained results. We hypothesize that a social robot displaying a behavior in accordance with the regulatory focus theory (i.e., promotion or prevention) can be used in different tasks (e.g., to play cognitive games, to motivate individuals to finish undesirable tasks) and with different populations (e.g., with children, with the elderly).

## 5. Conclusion and Future Work

In this paper, we have presented a study carried out with 42 participants in which a humanoid robot approached them in their own offices without being previously informed by the investigators that the interaction will take place (i.e., in the wild type of interaction). Out of the 42 individuals approached by the robot, only 29 interacted with it. The other 13, either avoided the robot or ignored it. In the interaction, the robot displayed one of two types of behaviors: promotion type or prevention type. The behavior was modeled on RFT that exists in the psychology literature. More specifically, in a promotion type of behavior the robot moved faster, spoke with a higher speech rate, and the message communicated was framed in the context of the desirable outcomes that can be obtained from successfully carrying out the task suggested by the robot. On the other hand, a prevention type behavior means lower moving speed, lower speech rate, and a message framed so as to show the undesirable outcomes that result from unsuccessfully pursuing a certain goal.

Our results show that the interaction time with a robot that matches the regulatory focus type of an individual is significantly longer than the interaction time with a robot that does not match the regulatory focus type of the individual. Therefore, we posit that the regulatory focus theory has to be considered when designing interactions between robots and humans.

Our future work will be focused on using a different RGB-D sensor so that the face detector can be more reliable. Furthermore, the approach behavior of the robot will be improved so that no intervention from the human operator is required. And finally, the French TTS will be installed on the robot and a speech recognition module will be used for a more natural dialog between the end users and the robot. Concerning the results of our work, we consider them as a basis for our future work. We plan on doing more in the wild experiments in order to test how the two robot behaviors (i.e., promotion and prevention) can be used in HRI.

## Data Availability Statement

The datasets generated for this study are available on request to the corresponding author.

## Ethics Statement

The studies involving human participants were reviewed and approved by ENSTA Paris. The patients/participants provided their written informed consent to participate in this study.

## Author Contributions

RA and S-DC contributed to the design of the experiment, to run the experiment, data analysis, and writing the article. AT contributed to the design of the experiment, to run the experiment, and writing the article.

## Conflict of Interest

The authors declare that the research was conducted in the absence of any commercial or financial relationships that could be construed as a potential conflict of interest.

## Footnotes

^1^http://tiago.pal-robotics.com/

^2^http://wiki.ros.org/navigation?distro=indigo

^3^http://wiki.ros.org/gmapping

^4^https://www.gimp.org

^5^http://www.fromtexttospeech.com

^6^https://micahallen.org/2018/03/15/introducing-raincloud-plots/
